# The Antarctic Weddell seal genome reveals evidence of selection on cardiovascular phenotype and lipid handling

**DOI:** 10.1038/s42003-022-03089-2

**Published:** 2022-02-17

**Authors:** Hyun Ji Noh, Jason Turner-Maier, S. Anne Schulberg, Michael L. Fitzgerald, Jeremy Johnson, Kaitlin N. Allen, Luis A. Hückstädt, Annabelle J. Batten, Jessica Alfoldi, Daniel P. Costa, Elinor K. Karlsson, Warren M. Zapol, Emmanuel S. Buys, Kerstin Lindblad-Toh, Allyson G. Hindle

**Affiliations:** 1 Broad Institute of MIT and Harvard, 7 Cambridge Center, Cambridge, MA 02142 USA; 2 Anesthesia Center for Critical Care Research, Department of Anesthesia, Critical Care and Pain Medicine, Massachusetts General Hospital, Harvard Medical School, 55 Fruit Street, Boston, MA 02114 USA; 3 Lipid Metabolism Unit, Center for Computational and Integrative Biology, Massachusetts General Hospital, Harvard Medical School, 55 Fruit Street, Boston, MA 02114 USA; 4 Institute of Marine Sciences, Department of Ecology and Evolutionary Biology, University of California Santa Cruz, 130 McAllister Way, Santa Cruz, CA 95060 USA; 5 Program in Bioinformatics and Integrative Biology, University of Massachusetts Medical School, Worcester, MA 01655 USA; 6 Science for Life Laboratory, Department of Medical Biochemistry and Microbiology, Uppsala University, Uppsala, 75237 Sweden; 7 School of Life Sciences, University of Nevada Las Vegas, 4505S Maryland Parkway, Las Vegas, NV 89154 USA

**Keywords:** Evolutionary biology, Cardiovascular biology

## Abstract

The Weddell seal (*Leptonychotes weddellii*) thrives in its extreme Antarctic environment. We generated the Weddell seal genome assembly and a high-quality annotation to investigate genome-wide evolutionary pressures that underlie its phenotype and to study genes implicated in hypoxia tolerance and a lipid-based metabolism. Genome-wide analyses included gene family expansion/contraction, positive selection, and diverged sequence (acceleration) compared to other placental mammals, identifying selection in coding and non-coding sequence in five pathways that may shape cardiovascular phenotype. Lipid metabolism as well as hypoxia genes contained more accelerated regions in the Weddell seal compared to genomic background. Top-significant genes were *SUMO2* and *EP300*; both regulate hypoxia inducible factor signaling. Liver expression of four genes with the strongest acceleration signals differ between Weddell seals and a terrestrial mammal, sheep. We also report a high-density lipoprotein-like particle in Weddell seal serum not present in other mammals, including the shallow-diving harbor seal.

## Abstract (web summary)

The Antarctic Weddell inhabits one of the most extreme environments on Earth. Comparative genomics with close relatives reveals the specific genetic adaptations for cardiovascular and fat metabolism that enable it to thrive.

## Introduction

The Weddell seal (*Leptonychotes weddellii*) is a highly specialized diving mammal occupying a unique ecological niche within the Antarctic sea ice. Weddell seals feed at ocean depths, but their resting and breeding areas are on the ice surface^1^. The species also meets the challenges of maintaining endothermy in extremely cold air temperatures that can plummet to −80 °C with high winds^2^. The morphological and physiological traits of the Weddell seal are likely evolutionary adaptations that allow this species to thrive as the world’s southernmost mammal^3^.

Weddell seals are particularly well adapted for deep diving. They experience profound bradycardia^4,5^, hypoxemia^6,7^, and peripheral vasoconstriction^8,9^ during dives lasting up to 90 min and feeding at ocean depths of up to 1000 m. Large oxygen stores in blood and muscle are a key feature of this exceptional breath-holding ability^10^. Preparations of isolated seal tissues also demonstrate innate resistance to hypoxia^11,12^. Thus, cell specializations that protect against low oxygen injury^13^ are important for diving ability. These are likely coupled with additional metabolic mechanisms that reduce energy expenditures during submergence^14–16^.

Modulation of lipid metabolism emerges as another key physiological feature of this species^17,18^. Like other pinnipeds, Weddell seals possess a thick specialized subcutaneous insulating blubber layer necessary for thermoregulation^2,19^ and which remains a metabolically active source of fuel during fasting periods associated with molting^20^ and lactation^21^. Evidence of the molecular specializations needed to rapidly mobilize, transport, and package maternal blubber energy stores into fat-rich milk (~48% lipids in Weddell seals^22^) will provide a deeper understanding of marine mammal metabolism.

Advances in genomics have rapidly expanded the number of whole genomes available for comparison across species^23^, allowing the systematic interrogation and understanding of both protein and regulatory changes through evolutionary events^24–26^. For pinnipeds, such analyses may enable discovery of specific genetic strategies facilitating cell and animal survival at low oxygen pressures and the absence of cardiovascular diseases despite hyperlipidemia, which may have indications for human medicine. Several pinniped genomes are now available^27–30^, however, functional genomics studies for marine mammals have centered primarily on cetaceans^31–34^. Despite clear phenotypic convergence among marine mammals (e.g., hydrodynamic body shapes and extended breath-holding abilities occurring in pinnipeds, cetaceans, and sirenians), analyses of molecular convergence have revealed only limited similarities among major proteins of interest^31,35–37^.

The Weddell seal genome is of particular interest for further investigation, as this species is one of the physiologically best-studied deep-diving marine mammals, providing systems for functional validation of genomic results. In addition, uncovering the genomic features that define this species may help us understand their sensitivity or resilience to future changes in the Southern Ocean ecosystem. To investigate the evolutionary history of the Weddell seal phenotype at the genome-wide level, we generated the first Weddell seal genome assembly (LepWed1.0) and a corresponding high-quality annotation, then performed comparative genomic analyses. We focused on gene family expansion and contraction, diverged sequence (acceleration), and evidence of positive selection on protein-coding sequences in the Weddell seal genome. To complement genome-wide analyses, we also specifically evaluated genes known to relate to hypoxia signaling and lipid metabolism, which are physiologies of interest in the Weddell seal.

## Results

### Genome assembly and annotation

De novo assembly using ALLPATHS-LG resulted in a genome assembly (82-fold read depth coverage) with a total ungapped length of 2.22 Gb (3.16 Gb including gaps), 16,711 scaffolds with N50 size of 0.90 Mb, 23,664 contigs with N50 size of 24 kb (Supplementary Table 1). The LepWed1.0 assembly (GenBank assembly accession: GCA_000349705.1, RefSeq assembly accession: GCF_000349705.1) contained 82.4% complete genes of the 4104 single-copy mammalian gene models evaluated by Basic Universal Single-Copy Orthologue analysis (including 43 genes that were duplicated). 11.9% of the remaining evaluated gene models were fragmented in the assembly, and 5.7% were missing.

RNA-seq data identified 109,900 transcripts, which included 64,408 high-confidence protein-coding isoforms. Based on RNA-seq, homology, and ab initio gene prediction (Supplementary Fig. 1), we predicted 22,027 protein-coding loci and 2649 functional non-coding genes in the Weddell seal. Our combined annotation improved the ab initio National Center for Biotechnology Information annotation, adding 2838 protein-coding genes and 2052 transcribed non-coding loci. As our annotation was designed to prioritize sensitivity, it also contains 35,105 additional loci which are either antisense to protein-coding genes or are currently unclassified.

### Comparative genomic analysis: gene family expansion and contraction

**Fig. 1:**
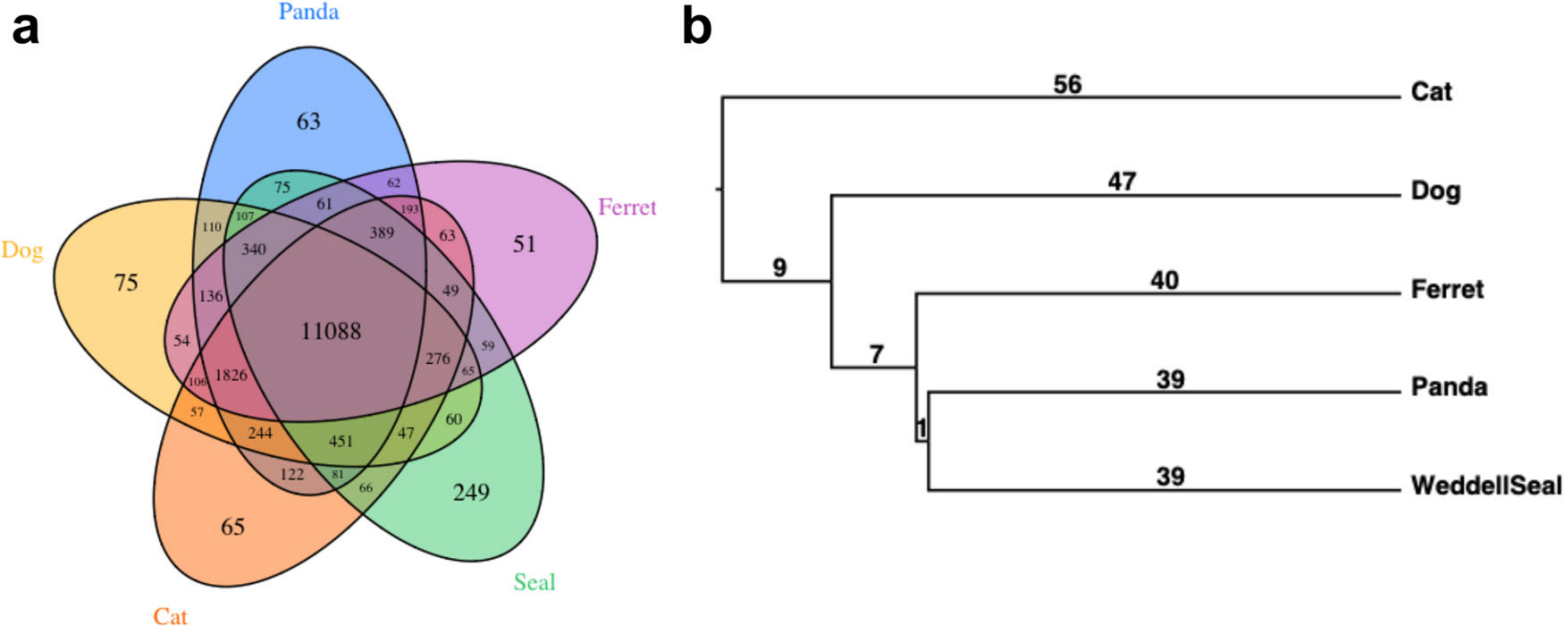
Gene family size analysis in the Weddell seal versus carnivores. **a** Orthogroup overlap among five carnivores, including the Weddell seal, identified by OrthoMCL. **b** A phylogenetic tree was applied to analyze gene family expansion/contraction in the seal lineage with CAFE; branch lengths (millions of years) were inferred from TimeTree.

16,690 gene families were identified from Weddell seals and four terrestrial carnivore proteomes (Fig. 1, Supplementary Table 2). The Weddell seal had a similarly sized proteome to other carnivores (Supplementary Table 2) but had the highest number of gene family contractions and expansions (35 significantly expanded gene families and 25 significant contractions; Supplementary Table 3). Top ranking (i.e., most significant) gene families contracted in the seal were cationic amino acid transporters and dimethylamine monooxygenases, and 14 of 25 contracted families were olfactory receptors (Supplementary Table 3). Top ranking expanded gene families included an immunoglobulin light chain, thyroglobulin, and dynein heavy chain proteins (Supplementary Table 3). Fourteen expanded gene families contained only Weddell seal proteins, pointing to the possibility that seal-specific genes are important in defining this species’ phenotype. Using Basic Local Alignment Search Tool, we identified these seal proteins and mapped them to gene families containing 60S ribosomal proteins, cytokines, calcium-binding proteins, histones, and ubiquitin proteasome (Supplementary Table 3). The high rate of protein-coding gene family expansions in the Weddell seal is consistent with a previous analysis of microRNA family size showing high rates of gain and loss in the Weddell seal lineage versus dog^38^.

### Comparative genomic analysis: dN/dS ratio

129 genes appear under positive selection (ratio nonsynonymous to synonymous substitution rates; dN/dS>1) in Weddell seal (Supplementary Table 4), relative to 28 placental mammals (listed in Supplementary Fig. 2). Functional annotation of these genes identified pathway enrichments for protein ubiquitination, DNA repair, and angiogenesis. The three top-ranking proteins under positive selection in the seal (ranked by number of selection sites) were retinoic acid induced 14 (*RAI14*), kinesin family member 6 (*KIF6*), and phosphodiesterase 11a (*PDE11A*); each had >50 sites of positive selection (Supplementary Table 4).

### Comparative genomic analysis: accelerated regions

**Fig. 2:**
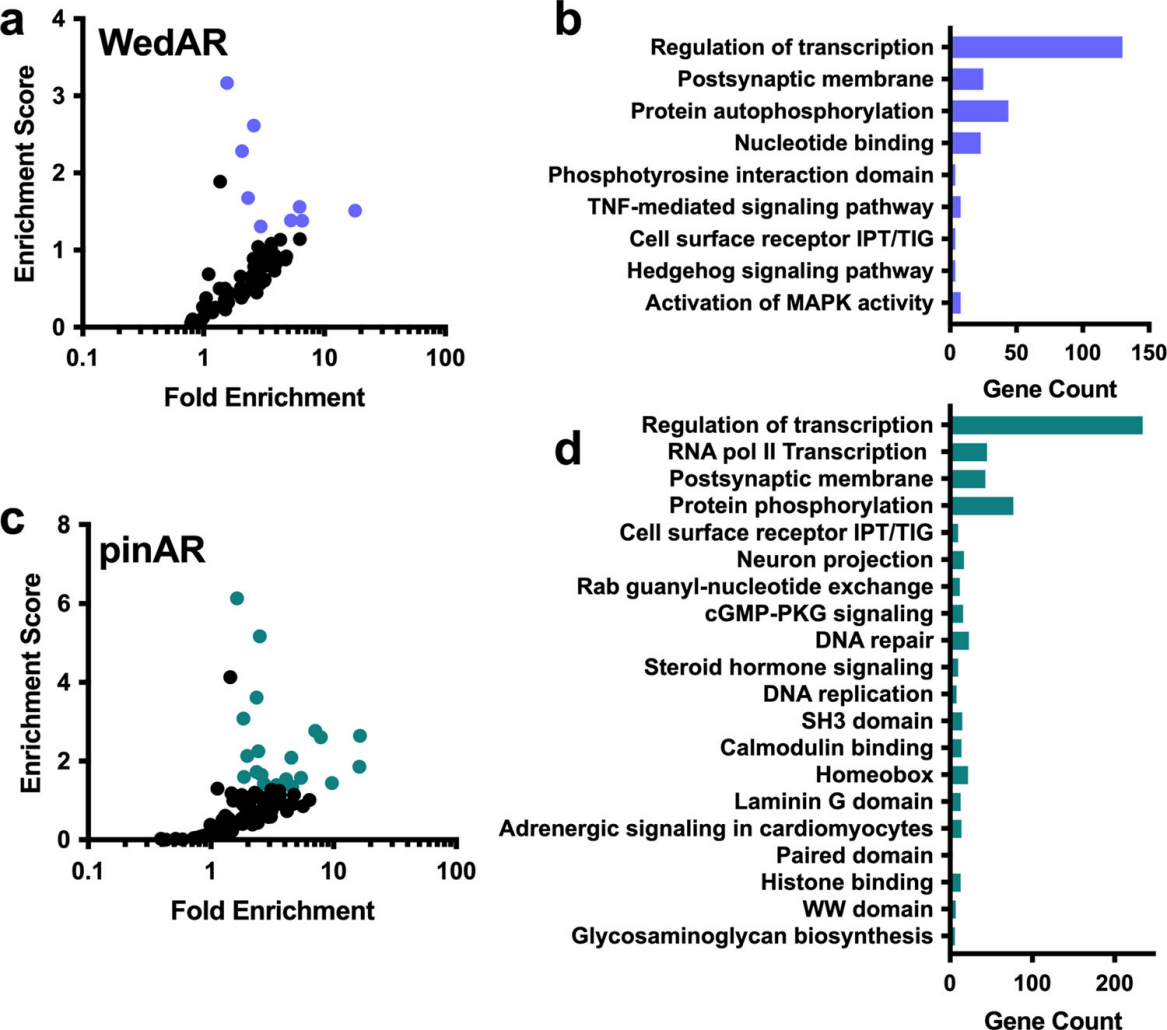
Significantly enriched pathways associated with genes containing Weddell seal or pinniped accelerated regions. **a** Pathway enrichments that met significance thresholds (an enrichment score >1.3 and a fold enrichment >1.5) for WedARs are noted in purple **b** pinARs are noted in green. The number of genes in each annotation cluster is also noted for **c** Weddell seal and **d** Weddell seal + walrus accelerated regions. Accelerated regions in species or branches of interest were identified relative to an alignment of 28 placental mammals.

We tested the conserved regions identified from the genomes of 57 placental mammals for divergence in both the Weddell seal and walrus and the seal alone. We identified 1471 pinniped accelerated regions (pinARs; mapping to 979 unique gene IDs) and 577 Weddell seal accelerated regions (WedARs; mapping to 514 unique Gene IDs). 264 accelerated regions (13%) were shared in both analyses. Both pinARs and WedARs are enriched for genes involved in transcriptional activities, protein kinase/phosphorylation signaling, and synaptic membrane components (Fig. 2; Supplementary Table 5), suggesting that transcriptional regulation and glutaminergic synapse functions have been commonly selected in pinnipeds. Enrichments in cyclic guanosine monophosphate signal transduction, calmodulin binding, and adrenergic signaling in cardiomyocytes also emerge from pinAR pathway analyses (Fig. 2c, d). The gene Patched 1 (*PTCH1*) had 9 accelerated regions all occurring in introns, which was the most WedARs of any gene in the dataset.

We also looked for pathways affected by genes with either WedARs or dN/dS selection sites. While only six individual genes (*AKAP1*, *DDX31*, *IQCH*, *PARD3B*, *PDE11A*, *WDR59*) had statistically significant scores in both metrics, we found 12 Ingenuity Pathway Analysis annotations that are enriched (*p* < 0.05) in both datasets: adrenomedullin signaling; axonal guidance signaling; C-C motif chemokine receptor 3 signaling in eosinophils; endothelin-1 signaling; extracellular signal-regulated kinase/mitogen-activated protein kinase signaling; molecular mechanisms of cancer; p21 activated kinase signaling; paxillin signaling; protein kinase A signaling; renin-angiotensin signaling; vascular endothelial growth factor family ligand–receptor interactions; vascular endothelial growth factor signaling.

While genes and enriched pathways identified by genome-wide analyses provide evidence for evolutionary selection that impacts gene regulation generally (transcription, translation, protein trafficking, and intracellular signaling), candidate genes and pathway enrichments specifically for cardiovascular regulation also arise from multiple analyses in the Weddell seal. These include a high positive selection on *KAI14*, *KIF6* (both indicated in cardiomyopathy), and *PDE11A* (vasoregulation), as well as pathway enrichments for the renin-angiotensin system and the endothelin-1 pathway, identified in both WedAR and dN/dS analyses. Heart rate regulation (cardiomyocyte adrenergic signaling) is also associated with pinARs.

### Hypothesis testing: selection on hypoxia signaling and lipid metabolism in Weddell seal

To identify genetic evidence of adaptive evolution for the traits of high interest, next, we looked for signals of selection that could be tied to known physiological and morphological specializations of pinnipeds generally, and the Weddell seal in particular. We tested for unusual enrichments of accelerated region signals in two gene lists: 84 genes implicated in hypoxia signaling; and 69 genes important in lipid metabolism. We found that acceleration signals in the Weddell seal genome were significantly stronger in both gene lists compared to genome-wide WedARs (mean false discovery rate (FDR) of 557,489 genome-wide split elements = 0.661). The mean FDR of 760 split elements annotated to the hypoxia signaling gene list was 0.647 (one-sided unpaired *t*-test *p* = 0.011, *t*-statistic = 2.291, df = 761 compared to the genome-wide FDR distribution) and the mean FDR of 2268 split elements annotated to the lipid metabolism gene list was 0.639 (one-sided unpaired *t*-test *p* = 4.9 × 10^−09^, *t*-statistic = 5.76, df = 2286). We performed the same analysis on pinARs, to evaluate whether the selections on hypoxia signaling and lipid metabolism are present in both the Weddell seal and walrus, compared to other mammals in this analysis. We found that lipid metabolism genes (mean FDR of 2226 split elements = 0.715, one-sided unpaired *t*-test *p* = 0.034, *t*-statistic = 1.83, df = 2245) but not hypoxia signaling genes (mean FDR of 737 split elements = 0.746, one-sided unpaired *t*-test *p* = 0.95, *t*-statistic = −1.67, df = 737.83), showed stronger acceleration signals compared to the genome-wide data for these two pinnipeds (mean FDR of 539,735 split elements = 0.735). These results identify lipid metabolism as a strongly selected pathway in both pinnipeds, whereas hypoxia signaling shows selection (i.e., a shifted distribution of FDR values for split elements in this gene list compared to genome-wide metrics) in the Weddell seal branch alone. Despite the strong selection signature for the lipid metabolism gene list in both the Weddell seal and the Weddell seal + walrus analysis, the top-significant genes are associated with obesity and not lipid transport. We did not detect a selection signature in the subset of lipid-transport genes within this list (WedAR mean FDR of 41 split elements = 0.694, one-sided unpaired *t*-test *p* = 0.859, *t*-statistic = −1.09, df = 40; pinAR mean FDR of 39 split elements = 0.725, one-sided unpaired *t*-test *p* = 0.345, *t*-statistic = 0.40, df = 38).

### Identification of candidate genomic elements in hypoxia signaling and lipid metabolism

**Fig. 3:**
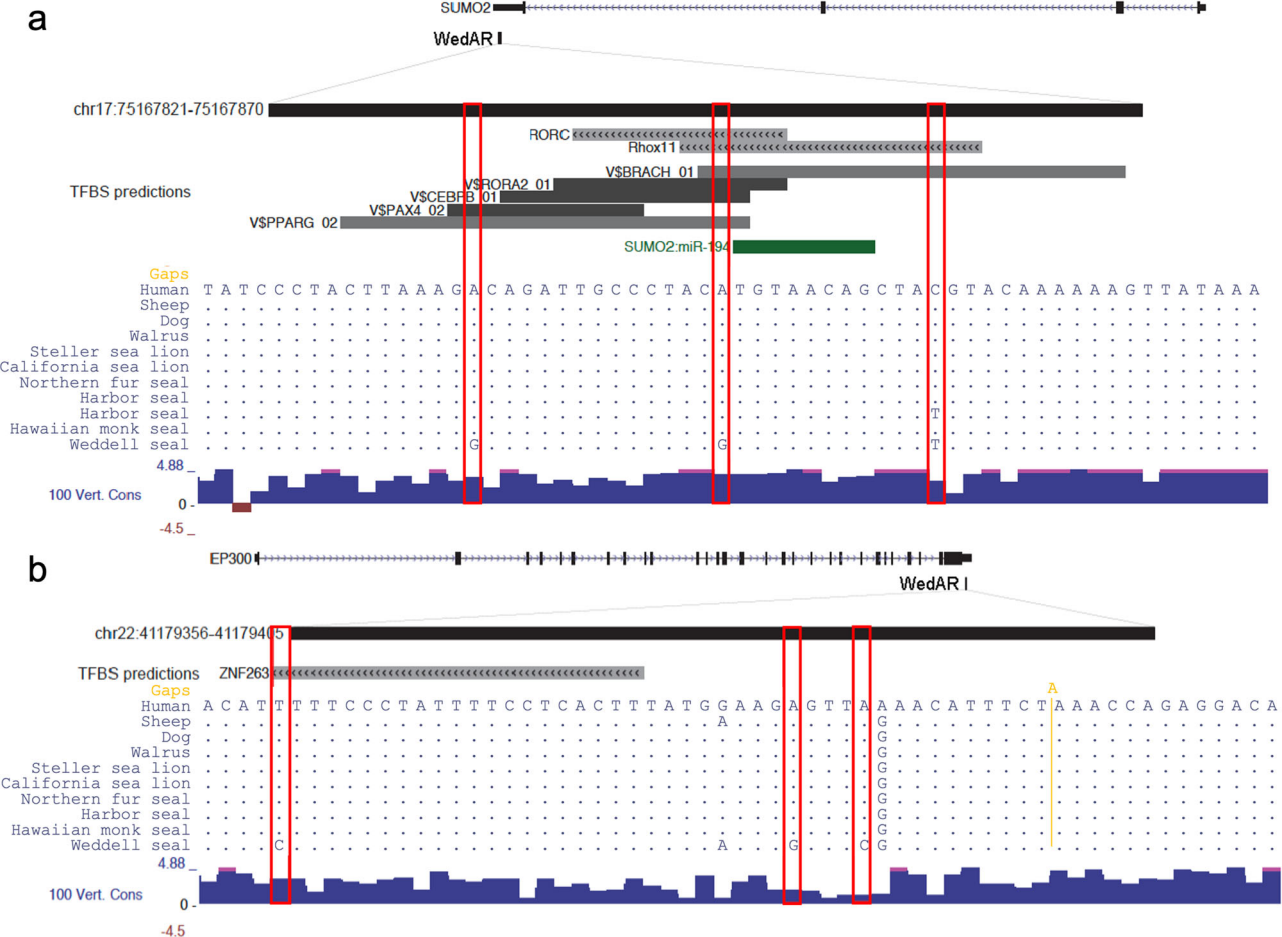
Location of Weddell seal-specific accelerated regions (WedARs) in candidate genes. **a** *SUMO2* and **b** *EP300*. Each panel contains a representation of the entire gene structure, with location of WedARs identified. For specific 50-bp regions containing WedARs, the complete human sequence, 100 vertebrate conservation track (100 Vert. Cons), and seal sequence differences are presented for sheep and dog, as well as all available pinniped sequences in GenBank (walrus, Steller sea lion, California sea lion, northern fur seal, harbor seal, Hawaiian monk seal, and Weddell seal). For *SUMO2*, two available harbor seal sequences differ within the WedAR region by a single base pair (first sequence: XM_032429487.1, second sequence: XM_032431333.1). Weddell seal-specific mutations are outlined in red bars. The location and identity of transcription factor binding sites (TFBS) within each WedAR are also noted.

To narrow down to specific genomic elements under adaptive evolution, we focused on the genes containing WedARs with the strongest acceleration signals (i.e., genome-wide-significance FDR < 0.1). *SUMO2* and *EP300* were identified as candidate hypoxia signaling genes under selection because they each contained a WedAR (for *SUMO2* this occurred in the 3′UTR, for *EP300* the accelerated region occurred in the 5′UTR; Table 1; Fig. 3). *SUMO2* and *EP300* are both key transcription factors, acting as a regulator and a co-activator of hypoxia-inducible factor (*HIF1A*), respectively. Alignment of these 50 bp WedARs against human as well as all other pinniped sequences available in GenBank reveal that these mutations are present in only the Weddell seal lineage, with the exception of one shared mutation of *SUMO2* in harbor seal (Fig. 3). Lipid metabolic genes showed a shift in FDR signal for both comparisons (Weddell seal genome alone, Weddell seal + walrus). Four significant WedAR elements (FDR < 0.1, Table 1) were identified in *ZBTB20* (implicated in glucose homeostasis and de novo lipogenesis) and one in *FTO* (encoding alpha-ketoglutarate dependent dioxygenase, also called obesity-related protein). A total of four pinAR elements were found in *FTO* (2 sites), *GRB14* (1 site), and *ABCA12* (1 site). Two pinARs were detected in *ZBTB20*, which were also two of four WedARs in the same gene, suggesting that *ZBTB20* is under selection in pinnipeds generally, but more so in Weddell seals (Table 1).

**Table 1:** Candidate genomic elements under selection (FDR < 0.1) in hypoxia signaling and lipid metabolism genes.

Gene	Genomic position of AR (hg38)	AR Type	AR FDR
*Hypoxia signaling*			
*SUMO2*	chr17:75167821–75167870	WedAR	0.005
*EP300*	chr22:41179356–41179405	WedAR	0.071
*Lipid metabolism*			
*ZBTB20*	chr3:114688654–114688703	WedAR	0
ZBTB20*	chr3:115100367–115100416	WedAR	0
*ZBTB20*	chr3:114496084–114496133	WedAR	0.038
ZBTB20*	chr3:114875113–114875162	WedAR	0.079
*FTO*	chr16:54059646–54059695	WedAR	0.047
*FTO*	chr16:54082989–54083038	pinAR	0.005
*FTO*	chr16:54062786–54062835	pinAR	0.028
*GRB14*	chr2:164498922–164498971	pinAR	0.030
ZBTB20*	chr3:115100367–115100416	pinAR	0.041
ZBTB20*	chr3:114875113–114875162	pinAR	0.098
*ABCA12*	chr2:214987361–214987410	pinAR	0.067

Accelerated regions were evaluated based on the placental mammal subset of the University of California Santa Cruz 100-way vertebrate alignment (hg38). False discovery rates (FDR) were calculated from phyloP empirical *p*-values. WedAR indicates accelerated regions identified in genes from the Weddell seal only. pinAR indicates accelerated regions identified in genes from the pinniped (Weddell seal + walrus) subtree. *Elements identified in both WedAR and pinAR. Gene symbols are in italics.

### Functional validation of candidate hypoxia signaling and lipid metabolism genes

To gain further confidence on adaptive evolution in hypoxia signaling and lipid metabolism, we tested functional comparisons of our candidate genes across related species. None of the seven WedARs identified in *SUMO2*, *EP300*, *ZBTB20*, and *FTO* overlap with predicted protein-coding sequences (Fig. 3, Supplementary Fig. 3), therefore, we hypothesized that these ARs could affect non-coding regulatory elements. Indeed, the sequences of all seven WedARs contain at least one known transcription factor binding motif conserved in mammals^39,40^ (Fig. 3, Supplementary Fig. 3). In addition, the WedAR in *SUMO2* contains a microRNA binding site (for *miR-194-5p*), which is predicted from seven-nucleotide seed regions conserved across mammals. Altered microRNA binding characteristics could affect the stability of the *SUMO2* mRNA.

**Fig. 4:**
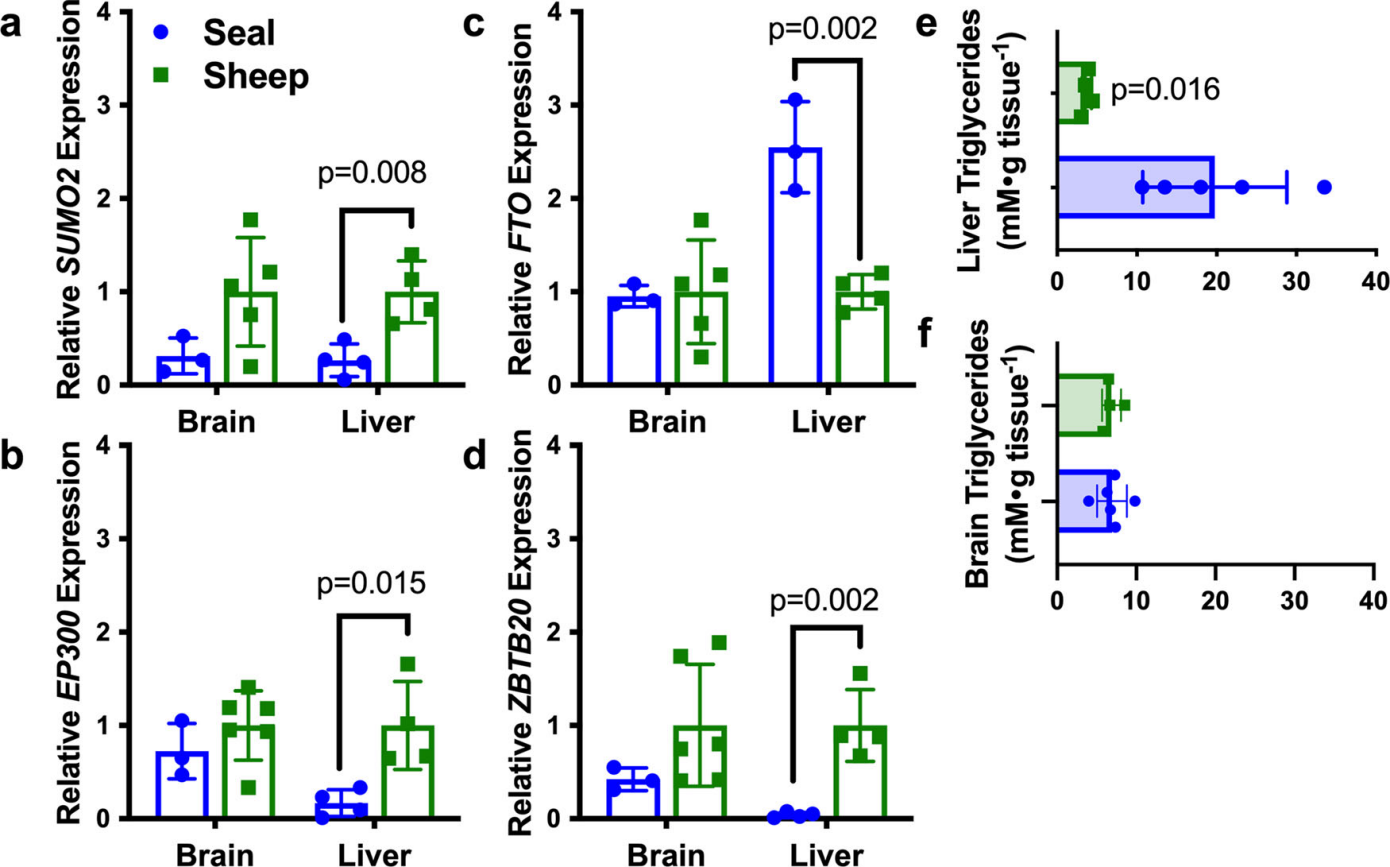
Transcript expression and triglyceride contents demonstrate tissue-specific differences between seals and sheep. Brain and liver expression of **a** *SUMO2*, **b** *EP300*, **c** *FTO*, and **d** *ZBTB20* were evaluated by qPCR in seals and in sheep. Total tissue triglyceride content were also assayed in **e** liver and **f** cerebral cortex of both species. Seal data are noted in blue circles, sheep data are noted in green squares throughout. *n* = 3 seal cerebral cortex samples were used for qPCR, *n* = 3–4 seal liver samples were used for qPCR. *n* = 4–6 sheep cerebral cortex samples were used for qPCR, *n* = 4 sheep liver samples were used for qPCR. *n* = 6 seal cerebral cortex homogenates, *n* = 5 seal liver homogenates, and *n* = 4 sheep sample homogenates from both tissues were used to assay total triglycerides. All replicates within each species comparison represent biologically independent samples. Bars represent means for each species and error bars represent st. dev. *P*-values are noted when comparisons between species were significant.

To examine tissue-specific and species-specific expression of these four genes of interest, we measured mRNA levels of *SUMO2*, *EP300*, *FTO*, and *ZBTB20* of Weddell seal and a terrestrial comparison species, sheep. Specifically, we focused on liver and brain, to compare tissues that we expect to rely on divergent lipid metabolism and hypoxia signaling strategies. The liver is the predominant site of lipoprotein, cholesterol, and phospholipid synthesis, as well as beta-oxidation, whereas the brain relies primarily on glucose as fuel. There are also differences in prioritization of perfusion and presumed hypoxia exposure between these two tissues during diving^9^. Expression of all four genes differed between species in the liver, but not in the brain (Fig. 4). *SUMO2*, *EP300*, and *ZBTB20* were lower in Weddell seal livers than in those of sheep. *FTO* expression was higher in the seal liver than in sheep (two-sided unpaired *t*-test, *t*-statistic = 5.962, df = 5, *p* = 0.002, Fig. 4c), which is consistent with 5-fold higher total liver triglycerides measured in the seal (Welch’s two-sided *t*-test, *t*-statistic = 3.964, df = 4.043, *p* = 0.016; Fig. 4e, f).

**Fig. 5:**
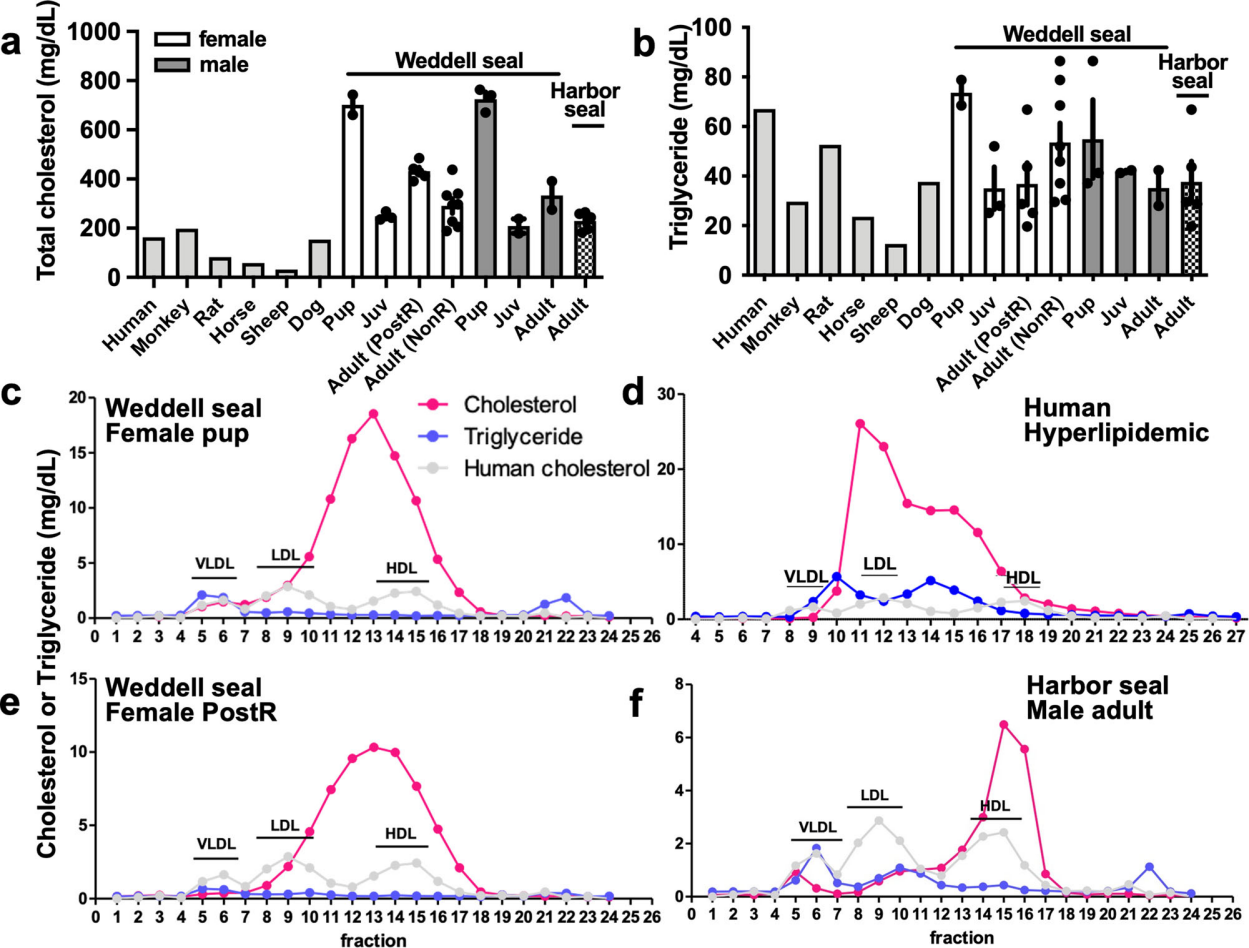
Analysis of lipid transport capacity in sera from a variety of mammals, including Weddell seal and harbor seal. Sera from a variety of species, along with Weddell seal samples, was separated by fast protein liquid chromatography and **a** total cholesterol and **b** total triglycerides was measured and summated in the fractions. The seal samples include free-ranging Weddell pups (3 male, 2 female) juveniles (2 male, 3 female), and adults (2 male, 5 post-weaning females (PostR), 8 non-reproductive females (NonR), and harbor seals (5 male). All samples plotted are independent biological replicates. Data are presented as mean with error bars representing s.e.m. for each seal group. The monkey, rat, horse, sheep, dog, and human samples were pooled sera from commercial sources (*n* = 1 of each species). Representative traces of plasma fractionation are shown for a Weddell seal pup (**c**), a post-weaning female (**e**), a harbor seal (**f**), and a hyperlipidemic human (**d**). Although the human sample has similarly high total cholesterol to the Weddell seal pup, the seal cholesterol is primarily carried by a more high-density lipoprotein-like particle, as compared to the high levels of low-density lipoprotein in the human sample. Serum from harbor seal males carry less cholesterol than the serum of Weddell seals. The gray trace in **c**, **d**, and **e** is cholesterol levels derived from purified human lipoprotein standards of very low-density lipoprotein (vLDL), low-density lipoprotein (LDL), and high-density lipoprotein (HDL).

Finally, we characterized lipid transport in the serum of Weddell seals compared to other mammals, including the shallow-diving harbor seal (Fig. 5). Weddell seals have distinctly higher total cholesterol in serum, compared to healthy individuals from other species. Total cholesterol was ~78% higher in Weddell seal pups and 40–60% higher in adult seals of both sexes than in the dog. Total cholesterol in harbor seal serum was also elevated relative to other mammals but was ~50% lower than in Weddell seal pups and was intermediate between adult Weddell seals and the dog (Fig. 5a). It is noteworthy that seal serum is capable of transporting large quantities of bound lipid, which is apparent in the high levels of circulating cholesterol in a high-density lipoprotein (HDL)-like lipoprotein particle in the Weddell seal that is present in lower-numbered fractions than expected for human HDL (Fig. 5c, e). Bound lipid in the serum of harbor seals is carried in a fraction consistent with HDL defined for humans (Fig. 5f). This is distinct from other mammals, particularly hyperlipidemic humans, who share similarly high total cholesterol to the Weddell seal pup, but carry cholesterol primarily bound to low-density lipoprotein (LDL) (Fig. 5d).

## Discussion

In this study, we present the first report of a genome assembly and annotation for the Weddell seal, the worlds southernmost mammal. Analyses to detect signatures of adaptive evolution in this species included genome-wide assessment of gene duplication (via gene family size) as well as selective pressure on both coding and non-coding sequences. Importantly, several phenotypic elements of the Weddell seal, namely hypoxia tolerance during deep-diving and high circulating cholesterol levels, exist well outside the bounds of terrestrial mammals. Targeted evaluation of gene lists associated with both of these phenotypes revealed candidate genes with high divergence in the Weddell seal compared to other placental mammals. Functional assessment of lipid transport capabilities of the blood of Weddell seals also identifies a potentially novel, HDL-like lipoprotein particle that is not detected in other species, including the harbor seal. The quality of the genome assembly presented here is by today’s standards quite low, with short scaffold and contig sizes. Selection analysis on the sequences of short contiguity that does not span full gene lengths can potentially result in bias due to missing bases. Nevertheless, the LepWed1.0 genome comes with a high-quality annotation, which provides great utility for genetic research of Weddell seal as well its related species.

Genes with positively selected sites in protein-coding sequence may reflect altered function. These genes could support the extreme cardiovascular phenotype of the Weddell seal, including an enlarged, flattened heart^41^. The three genes under the greatest positive selection in the dN/dS analysis are implicated in either cardiomyopathy (*RAI14*, *KIF6*) or vasoregulation (*PDE11A*). *RAI14* is associated with left ventricular mass^42^. *KIF6* is constitutively expressed in coronary arteries and other vascular tissues, with a role in coronary artery health. A human *KIF6* polymorphism has been linked to increased coronary heart disease risk^43^ and elevated plasma triglycerides^44^. Though Weddell seal positive selection sites occur at a different location than the human polymorphism, this highlights the possibility for selection on *KIF6* to modulate the cardiac phenotype of Weddell seals. Phosphodiesterases play a range of systemic and tissue-specific roles in the breakdown of second messengers (cyclic adenosine monophsophate and cyclic guanosine monphosphate), with downstream effects on nitric oxide signaling and vasoregulation. The control of peripheral vascular tone is a central element of the cardiovascular control evoked by diving mammals during submergence. As heart rate falls, regional selective peripheral vasoconstriction stabilizes blood pressure and limits the delivery of oxygenated blood throughout the body^9^. *PDE11A* was identified in this study as a candidate gene, having both positive selection sites in the Weddell seal and an intronic accelerated region shared in the seal + walrus branch. Moreover, other phosphodiesterases have previously been proposed to support the diving phenotype. Polymorphisms in *PDE10A* in the human Bajau (“sea nomads”^45^) have been linked to breath-holding capabilities for subsistence hunting, and *PDE10A* contains an intronic pinAR. Altered signal transduction within the nitric oxide-cGMP pathway via *PDE5A* has also been linked to Weddell seal diving physiology^46^.

Candidate genes examined in this analysis contain accelerated regions only in regulatory, non-coding sites such as transcription factor and microRNA binding motifs. Changes in regulatory elements are a primary mechanism by which phenotypic variation arises^47^. Evolutionary innovation through gene regulation can also facilitate tissue-specific adjustments to protein-based metabolic and enzymatic systems, whereas direct protein-coding modifications cannot. For human complex traits, >90% of associated single nucleotide polymorphisms fall outside of protein-coding regions^48^.

We did not detect any candidate genes or selection sites that were an exact match with previous genomic studies of marine mammals (reviewed in ref. ^49^), including a study that described pinniped-specific gene family size changes as well as positive selection on protein-coding sequences^29^. For the pinniped study, which focused on the Steller sea lion, spotted seal and northern fur seal (and included comparisons to Weddell seal and walrus), this lack of overlap may be due to the large differences in background species (9 other mammals including a marsupial, versus this study with 28 placental mammals) to evaluate dN/dS. Despite a lack of overlap in specific genes, we did find supporting evidence for convergent physiological phenotypes relative to other studies. For example, evolutionary selection on the renin-angiotensin signaling pathway in cetaceans includes mutations in the aminopeptidases *ANPEP* and *LNPEP*^33^, whereas we show positive selection on *ENPEP* in the Weddell seal. Each of these genes is involved in angiotensin conversion. We also note a similar pattern of gene family contraction in olfactory receptors in the Weddell seal, previously detected in cetaceans^50^ and in marine mammals overall^51^. Although gene family contractions may be an artifact of genes masked by this assembly, a high degree of gene loss is also a signal of evolutionary convergence in aquatic mammals^31^.

Endothelin-1 is a potent vasoconstrictive peptide with a potential role in regulating tissue perfusion during diving. Peripheral vasoconstriction in the face of intense bradycardia during submergence is imperative for the dive response and is thus a key component of the marine mammal diving phenotype. More than any other pathway^49^, selection on endothelin-1 signaling has been detected in marine mammals ranging from polar bears^52^ to mysticete whales^53^. As with renin-angiotensin signaling, candidate genes and type of selective pressures previously mapped to the endothelin-1 pathway appear species-specific and do not overlap with this analysis. Yet, the endothelin-1 pathway is enriched when gene outputs from selection and acceleration analyses are overlaid, implicating vasoregulation as a mechanism under selection in the Weddell seal.

Genetic adaptation to chronic hypoxia in high altitude populations of humans^54^, high altitude native mammals^55,56^, and even cetaceans^57^, has previously been linked to the HIF pathway, but not to *SUMO2* or *EP300*, each of which contains a Weddell seal accelerated region. When stabilized, either by external factors or lack of oxygen, HIFs translocate to the nucleus as transcription factors^58,59^. All three SUMO proteins are capable of *HIF1A* posttranslational modification, targeting near to the oxygen-dependent degradation domain and decreasing HIF transcription factor activity^60^. Low *SUMO2* mRNA detected in the seal liver would enhance HIF transcription factor activity, assuming decreased mRNA expression translates to reduced SUMO2 protein. Similarly, *SUMO 2/3* inhibits the effects of *EP300*^61^ (a *HIF-1* co-activator^62^) and lower *SUMO2* mRNA could mediate the HIF-stabilizing influences of *EP300*. Lower *EP300* mRNA expression in seal liver than in sheep is not consistent with increased HIF signaling, which would be expected in response to chronic intermittent hypoxia^63^. Yet, regulation of *HIF1A* is complex and it is not possible to entirely deconvolute the impact of the seal-specific modifications in the pathway within the constraints of our functional analyses, which examine baseline expression levels of regulatory genes but do not examine gene expression changes that could occur across the diving period^64^.

Based on the repeated exposure of pinnipeds to hypoxia for feeding during daily diving^65^, we hypothesize that Weddell seals demonstrate specialization to intermittent hypoxia via *HIF1A*, either through copy number, sequence differences, or interacting factors. We detected expression differences of genes that can interact with *HIF1A* between the seal and sheep, specifically in the liver, which experiences hypoperfusion on very long dives^8^. The ringed seal genome contains only one copy of *HIF1A*^66^ and our analysis of gene family size indicates the same for the Weddell seal. However, HIF1A amino acid sequence differences within carnivores do suggest pinniped specializations. Sequences from the Weddell seal (this study), ringed seal^66^, as well as harbor seal, Hawaiian monk seal, Steller sea lion, California sea lion, northern fur seal, and walrus (sequences obtained from NCBI) have two shared substitutions in *HIF1A* compared to other carnivores. One substitution occurs in the oxygen-dependent degradation domain and one lies directly upstream of the *EP300* binding site^67^. While these mutations could affect the signaling response to low oxygen, the functional outcome of these sequence changes and the differential expression of *HIF1A* modulating genes remain unclear. Previous functional studies point to an enhanced sensitivity of *HIF1A* in the tissues of ringed seals and elephant seals, relative to terrestrial mammals^66,68^ and it is reasonable to speculate that the intermittent nature of hypoxia exposure in pinnipeds (diving periods contrasted with extended haul-outs) may impose selective pressure on *HIF1A* sensitivity and response time course, rather than on changes in constitutive function.

Selection on lipid handling genes may explain the high body fat content observed in seals. *FTO*, *ZBTB20*, *GRB14*, and *ABCA12* were identified as candidate genes related to lipid handling in pinnipeds, based on the AR analysis. *FTO* polymorphisms have been linked to body mass and adiposity^69,70^. However, human *FTO* single nucleotide polymorphisms are found in intron1^69,70^, whereas the WedAR is located in the last intron, which contains a myeloblastosis family transcription factor binding element. *ZBTB20* functions include insulin regulation/glucose homeostasis and lipogenesis^71^. *GRB14* also regulates insulin signaling, inhibiting insulin receptor activity^72,73^. *GRB14* knock-down in mice improved both insulin signaling and de novo fatty acid synthesis in the liver^74^. *GRB14* polymorphisms have also been applied to gene scores that, along with *FTO*, predict human body mass index, body fat percentage, and cardiovascular risk^75,76^. *ABCA12* is critical for establishing the skin’s permeability barrier. In humans *ABCA12* loss-of-function mutation is associated with harlequin ichthyosis^77^, a neonatal skin disease marked by a profound expansion of corneocytes of the epidermal layer. Due to a defect in the transport and packaging of long-chain hydroxyceramides into the post-lysosomal secretory granules, these lipids are not incorporated into the intercellular lipid lamellar structure that is critical for establishing the epidermal permeability barrier. To date, *ABCA12* gain-of-function mutations have not been described in humans, but it may be that increased *ABCA12* function in pinnipeds could increase the permeability barrier of the epidermis as part of an adaption of the skin and underlying blubber layer to a cold marine environment.

The liver is a major site of lipid metabolic protein production and de novo lipogenesis. Lipid (triglyceride) content was 5-fold higher in seal liver than liver of a terrestrial comparison. Concordantly, liver-specific expression of *FTO* was higher in the Weddell seal versus sheep. Enhanced *FTO* expression is associated with increased adiposity in mice and can be induced, under certain conditions, by a high-fat diet (reviewed by ref. ^78^), both consistent with higher liver triglycerides of seals. Contrary to mice, which exhibit hepatic hypolipidemia in the absence of *ZBTB20*^71^, triglyceride content was higher in the liver of Weddell seals than sheep, despite lower *ZBTB20* mRNA expression in seals. However, *ZBTB20* is only indirectly related to lipogenesis in mice, and may have other functions in Weddell seals.

Weddell seals demonstrate high circulating cholesterol levels compared to other mammals, even other pinnipeds (Fig. 5; refs. ^79–81^), and selection on lipid handling and transport was hypothesized to underlie protection from cardiovascular disease that would likely develop in humans with similar risk factors. Notably, lipid fractionation profiles show a clear phenotypic difference between the Weddell seal and human in the amount of lipid carried and the apparent mass of the lipoprotein particle. Yet, this observation was not explained by direct evolutionary differences in genomic sequence of candidate lipid transport genes. Posttranslational protein modifications that alter lipoprotein molecular weight have been previously documented in the Weddell seal^82^ and could underlie the potentially unique lipid transport capabilities in this species. Even the harbor seal, which like the Weddell seal carries lipids primarily on an HDL-like particle, did not have a matching fractionation profile, highlighting mechanistic gaps that remain in our knowledge of pinniped physiology, which could be tied to environmental specializations.

The analysis of the Antarctic Weddell seal genome adds key information to our understanding of how these remarkable deep ocean divers have adapted to the cold polar environment and low oxygen pressures during diving. Our study shows multiple genomic selection signatures acting through different mechanisms, via controlling gene dosage (gene family size), protein-coding changes (dN/dS ratio), and non-coding regulatory changes (accelerated regions). Important in interpreting these signatures are the input datasets; the multi-species alignments used here in genome-wide analyses did not incorporate newly emerging genomes from other pinniped species (with the exception of the walrus genome in one analysis), therefore the power of this study to make conclusions about species-specific and pinniped family level evolutionary events is limited. We restrict our conclusions to identifying genes with signatures of evolutionary selection in the Weddell seal relative to other placental mammals, detecting genes under positive selection that are enriched for DNA binding and transcription factors, along with other regulatory functions affecting protein modifications and intracellular targeting. Our study also identifies evolutionary selection that could affect the cardiovascular system, regulation of hypoxia signaling and lipid synthesis, and transport mechanisms. These findings provide new targets for further exploration at the genetic and genomic levels, to gain insight into the specializations of this species to life in an extreme environment.

## Methods

### Sample collection and sequencing the Weddell seal genome

All Weddell seal handling and tissue collection were conducted under scientific authorizations from the National Marine Fisheries Service (NMFS 87-1851 & 19439) and the Antarctic Conservation Act. Tissue samples were collected at necropsy from Weddell seals in Erebus Bay, Antarctica, then snap-frozen for transport, to be used for genome sequencing as well as RNA-seq for improving the genome annotation. High molecular weight genomic DNA was extracted from the liver of one female Weddell seal using a Qiagen DNA kit with Genomic Tips and used to construct 180 bp paired-end fragment libraries (>45X coverage), 3 kb jumping libraries (>45X coverage), and 40 kb FOSSILLs (~1X coverage)^83^. These libraries were sequenced on Hi-Seq Illumina machines, producing 101 bp reads, used for genome assembly. Transcriptomes were sequenced from RNA collected from the heart (left ventricle), muscle (*longissimus dorsi*), lung and placenta from other Weddell seals in the region (a single sample for each tissue). RNA-seq libraries, used for genome annotation, were constructed from total RNA (isolated using Trizol) via the Illumina TruSeq Stranded mRNA library prep kit and sequenced on an Illumina Hi-Seq 2000 (paired-end reads 2×101) to a depth of 50 million reads per library.

### Sequence assembly, alignment, annotation, and evaluation

The Weddell seal genome was de novo assembled by ALLPATHS-LG software^84^, then annotated using a custom pipeline that combined a liftover of the pre-existing dog genome annotation^85^, a homology-based annotation produced by the National Center for Biotechnology Information, and Cufflinks-assembled RNA-seq data from four tissues. The annotation workflow is shown in Supplementary Fig. 1. We applied Basic Universal Single-Copy Orthologue analysis to assess the degree of annotation completeness^86^. This approach evaluates gene models from a genome of interest relative to 4104 gene models highly conserved across mammals.

### Comparative genomic analysis

To identify genomic evidence of adaptive evolution in the Weddell seal, we investigated three signatures of genome-wide selection: (1) gene family expansion and contraction, implicating selection on gene copy number; (2) dN/dS ratios, measuring selective pressure on protein sequence; and (3) accelerated regions, indicating normally constrained coding and non-coding sequences with elevated divergence in the Weddell seal.

### Gene family detection

We used the carnivore genomes available in Ensembl to calculate and analyze differences in gene family size, based on protein orthologs identified by OrthoMCL^87^ (v2.0.9, using default parameters). Four carnivore proteomes were obtained from Ensembl (dog, CanFam3.1; cat, Felis_catus_6.2; ferret, MusPutFur1.0; and panda, AilMel1), and splice versions were removed from each proteome with 19,343–19,910 proteins retained in each dataset (Supplementary Table 2). For the Weddell seal proteome, we included 19,694 high-confidence proteins that were identified by the following procedure. For each transcript in our annotation, we used TransDecoder (v1.0) to identify open reading frames and evaluate their likelihood of encoding a protein sequence. Transcripts with complete open reading frames that ended more than 55 bp prior to the end of the expressed sequence were marked as nonsense-mediated decay candidates and were not used further. 1392 genes had only nonsense-mediated decay candidate transcripts and were therefore excluded. For the remainder we selected a single transcript per gene to input to OrthoMCL using the following criteria, in order of importance: (1) Likely to be protein-coding (contains an open reading frame); (2) Open reading frame completeness; (3) Major isoform by expression level based on RNA-seq data. When no single gene was clearly indicated, we allowed multiple transcripts to be input into OrthoMCL, to maximize the chances that gene families would be assembled. Prior to analysis of gene family sizes, we retained only the single gene representative that was mapped to the largest gene family.

### Gene family expansion analysis

Gene duplication is an important evolutionary avenue for acquiring new gene functions, as multiple copies can remove constraint to retain original function. To detect gene duplication events, we identified expansion (along with contraction) in Weddell seal gene families compared to dog, cat, ferret, and panda using CAFE v3.1^88,89^ using gene family orthologs detected in our dataset. CAFE provides a phylogenetically informed test of changes in gene family size that infers the rate and direction of these changes from predicted common ancestral family sizes. Calculation of the predicted expansions/contractions in Weddell seals relative to the carnivore lineage was based on a phylogenetic tree with branch lengths inferred from TimeTree^44^. CAFE was implemented with 100,000 iterations, a *p*-value cutoff of 0.05 to determine family-wise expansion/contraction for each gene family and was calculated as a maximum likelihood value. Within altered-size gene families, CAFE identifies specific branches with low *p*-values for the transition from the ancestral family size, which are considered most likely responsible for altered gene family size overall. We selected unusually evolving gene families in the Weddell seal branch (*p* < 0.05 in seal but not in other branches) for further evaluation. To consider functional significance of changes in gene family sizes, we analyzed Gene Ontology terms associated with named genes within each orthogroup, i.e., the group of genes descended from a single gene in the last common ancestor.

### dN/dS analysis

The dN/dS ratio, representing nonsynonymous to synonymous substitution rates, provides a metric for the net balance among neutral, deleterious, and beneficial mutations on protein-coding sequences. Evidence of positive selection in Weddell seal protein-coding sequences was evaluated against 28 mammals. We used the VESPA (Very large-scale Evolutionary and Selective Pressure Analyses) pipeline^90^ to calculate dN/dS ratios and evaluate selective pressure on protein-coding regions of the Weddell seal genome. This pipeline facilitates ortholog identification, multiple sequence alignment, phylogenetic reconstruction, and assessment of codon-based models of evolution, and was implemented with default parameters. We collected the transcriptomes of 28 mammals from Ensembl (Supplementary Fig. 2), including human, dolphin, and platypus, and used our updated annotation for the Weddell seal to generate a predicted transcriptome that contained complete, likely protein-coding, major isoforms. We created a homologous protein-coding gene dataset using all-against-all Basic Local Alignment Search Tool within the VESPA pipeline, then grouped sequences across species using the ‘best reciprocal’ function. We generated amino acid replacement models for each multiple sequence alignment (ProtTest3^91^) and used MrBayes (v3.2.6^92^) for phylogenetic reconstruction based on sequence data. Only sequence groups comprised of >7 species and including a single-copy seal ortholog were retained and were evaluated for site-specific selective pressure with codeml in PAML^93^, implemented within VESPA using default parameters^90^. Alignments and positive selection sites identified by codeml branch-site modelA results were visualized in ClustalX and manually validated.

### Accelerated region analysis

Sequence conservation across the phylogenetic tree is one of the best indicators of functional importance for a genomic region. When conserved regions differ from the expected sequence in specific species or lineage, it indicates a shift in evolutionary pressure specific to that species or subtree, representing either positive selection or relaxation of purifying selection. Genomic sequence with accelerated divergence in the Weddell seal was detected using 57 placental mammals present in the University of California Santa Cruz 100way vertebrate genome alignment (versus hg38; species list is included as Supplementary Table 6). To identify genomic coding and regulatory regions under positive selection we searched for accelerated regions in pinniped genomes (Weddell seal, LepWed1.0; walrus, odoRosDiv1) available in this multi-species alignment against conserved elements across species. Both the seal and walrus (*Odobenus rosmarus divergens*) inhabit high latitude marine environments and rely on a large, metabolically active subcutaneous blubber layer. Yet, these species differ in their diving behavior, with submergence duration and hypoxia tolerance of the Weddell seal far exceeding the walrus^65,94–96^. We ran the same analysis on the Weddell seal branch alone, as well as the two pinnipeds in this alignment combined (Weddell seal plus walrus) to correlate genes containing accelerated regions in just the Weddell seal versus those present in both species (but not other mammals) with the similarities and differences in the species’ physiology.

We regularized sequence lengths into 50 bp fragments. We included a phastCons model, also obtained from the University of California Santa Cruz genome browser and trimmed to placental mammals, as a neutral model of evolution. We then used phyloP implemented in the RPHAST package (mode = “ACC”)^97^ to obtain a series of likelihood ratio tests and empirical *p*-values on these 50 bp elements (based on 100,000 simulations) to identify elements with accelerated divergence. We performed likelihood ratio tests comparing the pinniped sequences to the conservation sequence track for placental mammals, evaluating 539,735 and 557,489 elements for pinniped (seal + walrus) and Weddell seal, respectively. After FDR correction^98^, 50 bp regions with corrected-*p* < 0.05 were identified as having a change in evolutionary rate specific to the species or subtree of interest, and were considered accelerated regions for downstream analysis. Accelerated regions were annotated with gene identities based on hg38.p11, if their genomic coordinates fell within 10 kb upstream or downstream of any given gene’s coding domain sequence (thus, accelerated regions could map to multiple genes).

### Gene set enrichment tests

We identified enrichment of gene pathways with DAVID Functional Annotation^99,100^ (using the human genome as the background and using an EASE score cut-off of *p* < 0.05 for significance) for proteins identified by codeml to be under positive selection in Weddell seals, and for genes associated with either pinniped or Weddell seal accelerated regions. For enriched annotation clusters (enrichment score >1.3), we report non-redundant cluster names according to the top-ranked KEGG Pathway or Gene Ontology term, if available. Ingenuity Pathways Analysis comparison analysis (Qiagen) was applied to identify pathways that were enriched in both accelerated region and dN/dS datasets.

### Hypothesis testing: accelerated regions in hypoxia signaling and lipid metabolism/transport genes

To complement the unbiased search of genes/genomic elements under selection by genome-wide comparative analyses, we also directly tested the hypotheses that hypoxia signaling and lipid metabolism are under selection in Weddell seals. We generated two gene lists, one that is relevant to hypoxia signaling responses (84 genes contained in the Human Hypoxia Signaling Pathway qPCR array, PAHS-032A, SABiosciences) and the other to lipid metabolism (69 genes curated from the literature; Supplementary Table 7) and examined gene list-specific accelerated regions that were present in both pinnipeds (Weddell seal + walrus) and in the Weddell seal alone. We obtained 760 (Weddell seal) and 737 (pinniped) 50 bp elements that are annotated to hypoxia signaling genes, and 2268 (Weddell seal) and 2226 (pinniped) elements that are annotated to target lipid metabolism genes. We then compared the distribution of the corrected *p*-values of the 50 bp elements (potential accelerated regions) that are annotated with target gene lists with the corrected *p*-values of all 50 bp elements genome-wide. We further evaluated a subset of the lipid-related gene list related specifically to lipid transport (Supplementary Table 7) and obtained 41 elements annotated to Weddell seal target lipid transport genes and 39 elements annotated to Weddell seal + walrus.

### Gene expression assays

To seek a corresponding functional output for target genes revealed by accelerated regions analyses, we compared regional gene expression in adult Weddell seal liver and brain (cerebral cortex). We also obtained cerebral cortex and liver samples from sheep (*Ovis aries*) as the closest terrestrial comparison from which we could obtain high-quality tissues (Supplementary Fig. 2). Total RNA was isolated in Trizol from these tissues. Transcript expression was determined by RT-qPCR using Fast SYBR Green Master Mix (Life Technologies). SYBR Green oligonucleotide primer pairs are listed in Supplementary Table 8. Genes of interest were normalized to 18S ribosomal RNA in each sample, amplified by human Taqman primers (Hs03003631, FisherScientific) for both species, using the deltaCT method.

### Lipoprotein, cholesterol, and triglyceride analysis

As a functional assessment of lipid handling, a physiology of interest in the Weddell seal, we also characterized serum lipoproteins and serum lipid content in the seal compared to a variety of mammals. Specifically, the seal serum samples included free-ranging Weddell pups (3 male, 2 female) juveniles (2 male, 3 female), and adults (2 male, 5 post-weaning females (PostR), 8 non-reproductive females (NonR), and harbor seals (5 adult males, obtained from a different study under authorization NMFS 18662). Seal samples were compared against monkey (#17305), rat (#17298), horse (#15398), sheep (#17116), and dog (#16947), which were obtained from commercial sources (Innovative Research, MI), as well as human male pooled sera (Sigma #026k067), and an anonymous hyperlipidemic serum sample from the Massachusetts General Hospital Lipid Metabolism Unit. Circulating lipoproteins were separated from pre-warmed (5 min at 37 °C) and filtered sera (polyvinylidene fluoride 0.45 μm membrane filter) by fast protein liquid chromatography gel filtration on a Superose 6 Precision Column 3.2/30 at 4 °C (GE Healthcare). Eluted serum fractions were monitored at 280 nm absorbance, with a constant flow of 40 μl min^−1^. We collected fractions beginning 18 min after sample injection, then determined cholesterol (TR13421, Thermo Fisher Scientific) and triglyceride content (TR0100-1KT, Sigma-Aldrich) in each fraction. The areas under the curve for vLDL, LDL, and HDL (very low, low, and high-density lipoproteins) were determined in comparison to a standard of known amounts of human vLDL, LDL, and HDL run in parallel. Total triglycerides were also assayed in the livers and cerebral cortex of adult Weddell seals (*n* = 5 liver, *n* = 6 brain) and sheep (*n* = 4 liver, *n* = 4 brain; EnzyChrom ETGA-200, BioAssay Systems).

### Candidate gene alignments and annotation of regulatory sites

We aligned a selection of 50 bp regions containing WedARs, contained within genes of interest, against other mammals to confirm our findings of accelerated divergence in the Weddell seal genome. We compared the Weddell seal sequence against human, dog, and sheep sequences identified in the University of California Santa Cruz genome browser, and manually appended the corresponding 50 bp sequences for other pinnipeds (harbor seal, Hawaiian monk seal, Steller sea lion, California sea lion, northern fur seal, walrus, available in GenBank). Gene models were captured from the University of California Santa Cruz genome browser. To identify putative regulatory sites in the Weddell seal sequences, we also mapped potential regulatory sequence locations, aligned on the human genome (hg19), that were available as tracks in the University of California Santa Cruz genome browser. Transcription factor binding sites were identified from the HMR Conserved Transcription Factor Binding Site (Z-score cutoff set to 0) and the JASPAR Transcription Factor Binding Site database. Predicted microRNA binding sites were identified from TargetScanHuman 7.2.

### Statistics and reproducibility

To evaluate selection on genes of interest (hypoxia signaling or lipid metabolism-related genes), we compared the distribution of corrected *p*-values of the 50 bp elements (potential accelerated regions) that were annotated with target gene lists, with genome-wide corrected *p*-values (all 50 bp elements), using a one-sided unpaired *t*-test. Normalized transcript expression for candidate genes (*SUMO2*, *EP300*, *FTO*, and *ZBTB20*) in the liver and cerebral cortex were compared between seals and sheep using two-sided unpaired *t*-tests, without assuming consistent standard deviations between groups, and correcting for multiple tests using the Holm-Sidak method. Uncorrected *p*-values are reported in the text and in Fig. 4. We compared regional gene expression in adult Weddell seal liver (*n* = 3–4) and brain (cerebral cortex, *n* = 3) with corresponding gene expression in the sheep liver (*n* = 4) and cerebral cortex (*n* = 4–6). Total triglycerides were compared with a Welch’s two-sided *t*-test between species in the liver (*n* = 5 seal, *n* = 4 sheep) and brain (*n* = 6 seal, *n* = 4 sheep). All measurements were collected from distinct samples.

### Live vertebrates

This study complied with all relevant ethical regulations related to animal research. Tissue and serum collection from Weddell seals (*n* = 31 total animals) and tissue collection from sheep (*n* = 6 adult females in total), received ethical approval from the Massachusetts General Hospital Institutional Animal Care and Use Committee. Serum was collected from 25 live-handled Weddell seals (7 male, 18 female): 5 pups, 5 juveniles, and 15 adults.

### Reporting summary

Further information on research design is available in the Nature Research Reporting Summary linked to this article.

## Supplementary information

Supplementary Information

Description of Additional Supplementary Files

Supplemental Data 1–8

Reporting Summary

## Data Availability

The sequence datasets generated and analyzed in the current study are publicly available. The assembly is deposited into the National Center for Biotechnology Information GenBank Assembly database under accession # APMU00000000^101^. Raw transcriptome sequences used for genome annotation are deposited into the National Center for Biotechnology Information Sequence Read Archive under accession # PRJNA474945^102^. The genome annotation^103^ (10.6084/m9.figshare.16654993) and source data for presented figures^104^ (10.6084/m9.figshare.16655062.v1) are available on FigShare. There are no restrictions on data availability. All other data are available on request from the corresponding author.
