# Supplementary material for: The Antarctic Weddell seal genome reveals evidence of selection on cardiovascular phenotype and lipid handling
Source: Commun Biol. 2022 Feb 17;5:140. doi: 10.1038/s42003-022-03089-2 (PMC8854659; doi:10.1038/s42003-022-03089-2)
Supplement: Supplementary file 1 — Supplementary Information [file 42003_2022_3089_MOESM1_ESM.pdf]

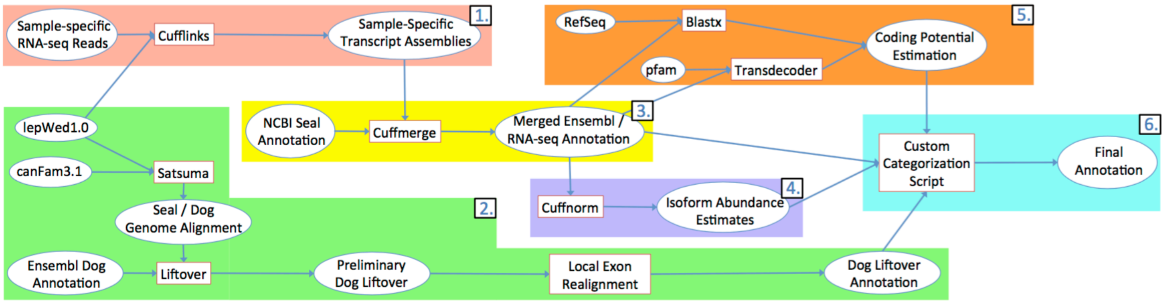

Figure S1. The Weddell seal genome annotation was constructed by the following six steps:

1. RNA-seq assembly: RNA-seq reads for each sample were aligned using Tophat and assembled into transcript files using Cufflinks, with one transcript file per sample.
2. Dog annotation liftover: The Weddell Seal genome and the Ensembl dog genome (CanFam3.1) were aligned using the syntenic aligner Satsuma to map the annotations from the dog genome onto the corresponding regions of the seal genome. The placement of dog annotations onto genomic loci in the sea genome fine-tuned using the local alignment program Rum.
3. Transcript Reconciliation: The four tissue RNA-seq assemblies were reconciled with Cuffmerge, which also integrated the NCBI *ab initio* annotation as a reference. When annotations were identified from both the dog annotation liftover and the Weddell seal annotation available at NCBI, we retained/prioritized the NCBI annotation.
4. Abundance estimation: Cuffnorm estimated normalized abundance values for each transcript across all samples.
5. Coding potential: As a proxy for coding potential, transcripts were compared to existing protein databases. A match in either Pfam or RefSeq was taken to indicate coding potential.
6. Categorization: A custom script read the annotation information into a database and categorizes the transcript loci as follows: high confidence protein coding (if RNAseq evidence); low confidence protein coding (if no RNAseq evidence); spliced anti-sense; unspliced anti-sense; lncRNA; other non-coding; and unclassified.

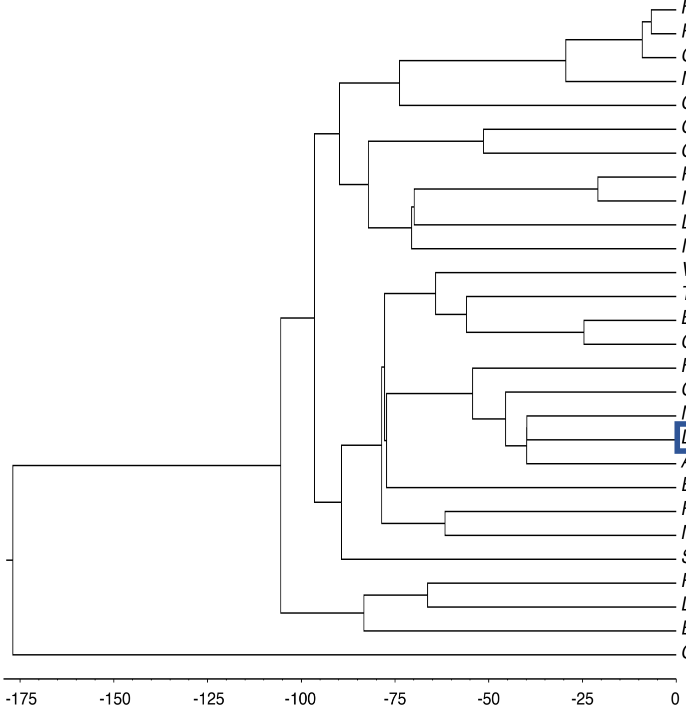

	Common Name	Assembly Version
Homo sapiens	human	GRCh38.p11
Pan troglodytes	chimpanzee	Pan_tro 3.0
Gorilla gorilla	gorilla	gorGor4
Macaca mulatta	macaque	Mmul_8.0.1
Otolemur garnettii	bushbaby	OtoGar3
Oryctolagus cuniculus	rabbit	OryCun2.0
Ochotona princeps	pika	OchPri2.0-Ens
Rattus norvegicus	rat	Rnor_6.0
Mus musculus	mouse	GRCm38.p5
Dipodomys ordii	kangaroo rat	dipOrd1
Ictidomys tridecemlineatus	squirrel	SpeTri2.0
Vicugna pacos	alpaca	vicPac1
Tursiops truncatus	dolphin	turTru1
Bos taurus	cow	UMD3.1
Ovis aries	sheep	Oar_v3.1
Felis catus	cat	Felis_catus_6.2
Canis lupus	dog	CanFam3.1
Mustela putorius	ferret	MusPutFur1.0
Leptonychotes weddellii	seal	LepWed1.0
Ailuropoda melanoleuca	panda	ailMel1
Equus caballus	horse	Equ Cab 2
Pteropus vampyrus	megabat	pteVam1
Myotis lucifugus	microbat	Myoluc2.0
Sorex araneus	shrew	sorAra1
Procavia capensis	hyrax	proCap1
Loxodonta africana	elephant	Loxafr3.0
Echinops telfairi	tenrec	TENREC
Ornithorhynchus anatinus	platypus	OANA5

Figure S2. List of species transcriptomes used to compare selective pressure in Weddell seal proteins using the VESPA pipeline. Phylogenetic tree indicating relationships between species was constructed with the TimeTree drawing feature (axis is millions of years).

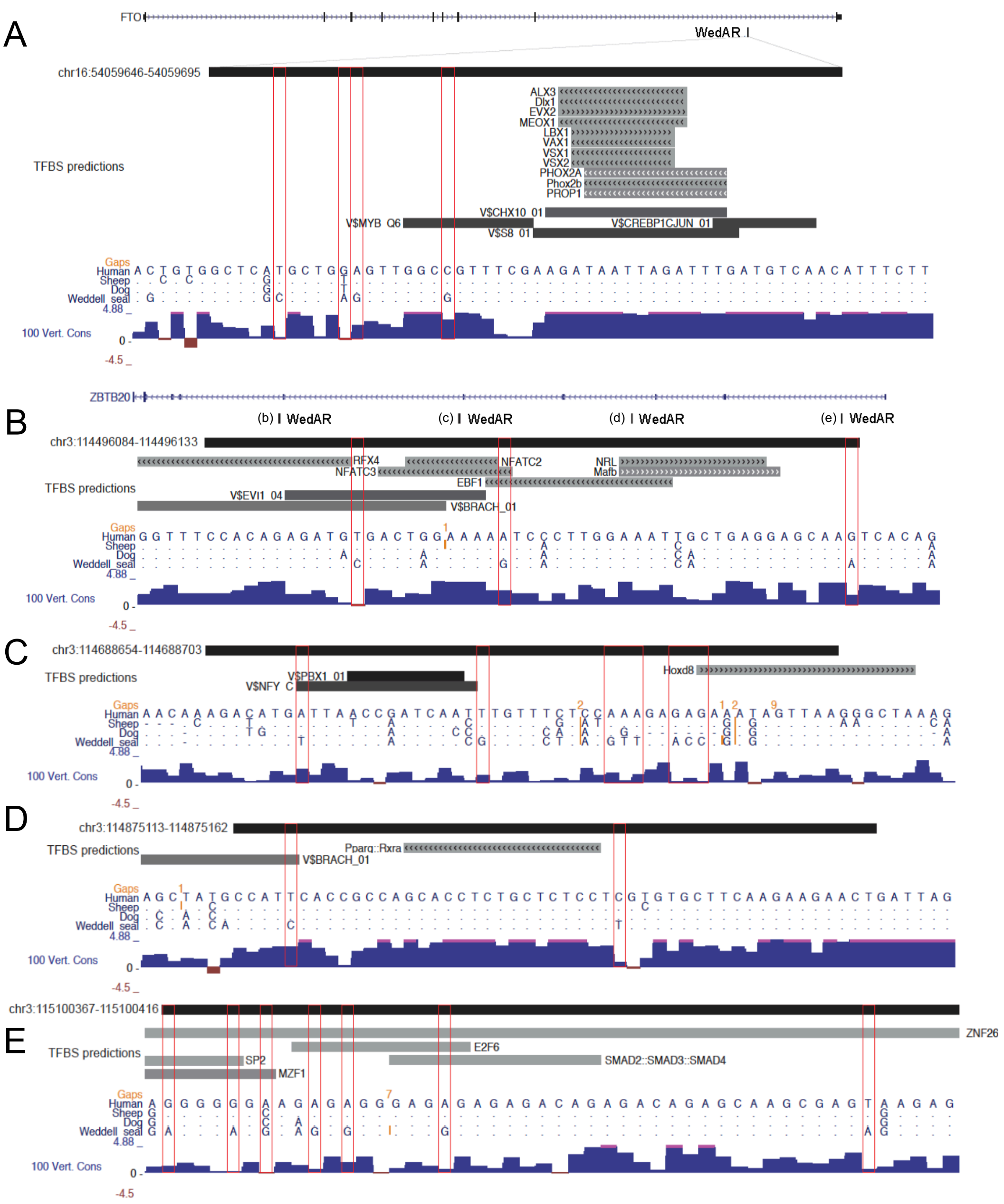

Figure S3. Location of Weddell seal-specific accelerated regions (WedARs) in candidate genes (a) *FTO*, and (b-e) *ZBTB20*. Each panel contains a representation of the entire gene structure, with location of WedARs identified. For specific 50-bp regions containing WedARs, the complete human sequence, 100 vertebrate conservation track (100 Vert. Cons), and seal sequence differences among sheep, dog and Weddell seal against the human sequence are included. Seal-specific mutations are outlined in red bars. The location and identity of transcription factor binding sites (TFBS) within each WedAR are also noted.
