# Supplementary material for: The Antarctic Weddell seal genome reveals evidence of selection on cardiovascular phenotype and lipid handling
Source: Commun Biol. 2022 Feb 17;5:140. doi: 10.1038/s42003-022-03089-2 (PMC8854659; doi:10.1038/s42003-022-03089-2)
Supplement: Supplementary file 2 — Description of Additional Supplementary Files [file 42003_2022_3089_MOESM2_ESM.pdf]

Description of Additional Supplementary Files

File name: Supplementary Data 1-8
Description:
Supplementary Data 1: Genome size data for pinniped representative genomes.

Supplementary Data 2: Gene families that showed expansion, no change, or contraction for carnivore species, based on the most likely assignments of ancestral gene family sizes.

Supplementary Data 3: Gene families that were expanded or contracted in the Weddell seal lineage, based on a stochastic birth and death model.

Supplementary Data 4: List of genes containing at least one positive selection site in Weddell seals.

Supplementary Data 5: Enriched pathways among genes containing accelerated regions.

Supplementary Data 6: Species list of 57 placental mammals included in accelerated region analysis.

Supplementary Data 7: List of 84 hypoxia and 69 lipid genes used for hypothesis testing and their genomic positions.

Supplementary Data 8: Primer sequences used for qPCR in Weddell seals and sheep.
