# Supplementary material for: The Antarctic Weddell seal genome reveals evidence of selection on cardiovascular phenotype and lipid handling
Source: Commun Biol. 2022 Feb 17;5:140. doi: 10.1038/s42003-022-03089-2 (PMC8854659; doi:10.1038/s42003-022-03089-2)
Supplement: Supplementary file 4 — Reporting Summary [file 42003_2022_3089_MOESM4_ESM.pdf]

nature portfolio

Corresponding author(s): Allyson Hindle

Last updated by author(s): Dec 22, 2021

Reporting Summary

Nature Portfolio wishes to improve the reproducibility of the work that we publish. This form provides structure for consistency and transparency in reporting. For further information on Nature Portfolio policies, see our Editorial Policies and the Editorial Policy Checklist.

Statistics

For all statistical analyses, confirm that the following items are present in the figure legend, table legend, main text, or Methods section.

n/a	Confirmed	
☐	☒	The exact sample size (*n*) for each experimental group/condition, given as a discrete number and unit of measurement
☐	☒	A statement on whether measurements were taken from distinct samples or whether the same sample was measured repeatedly
☐	☒	The statistical test(s) used AND whether they are one- or two-sided<br>*Only common tests should be described solely by name; describe more complex techniques in the Methods section.*
☒	☐	A description of all covariates tested
☐	☒	A description of any assumptions or corrections, such as tests of normality and adjustment for multiple comparisons
☐	☒	A full description of the statistical parameters including central tendency (e.g. means) or other basic estimates (e.g. regression coefficient) AND variation (e.g. standard deviation) or associated estimates of uncertainty (e.g. confidence intervals)
☐	☒	For null hypothesis testing, the test statistic (e.g. *F*, *t*, *r*) with confidence intervals, effect sizes, degrees of freedom and *P* value noted<br>*Give P values as exact values whenever suitable.*
☒	☐	For Bayesian analysis, information on the choice of priors and Markov chain Monte Carlo settings
☒	☐	For hierarchical and complex designs, identification of the appropriate level for tests and full reporting of outcomes
☒	☐	Estimates of effect sizes (e.g. Cohen's *d*, Pearson's *r*), indicating how they were calculated

Our web collection on statistics for biologists contains articles on many of the points above.

Software and code

Policy information about availability of computer code

Data collection	The Weddell genome assembly was produced with ALLPATHS-LG, and the annotation was produced using a suite of commercially available software (Tophat, Cufflinks, Satsuma, Rum, Cuffmerge, Cuffnorm). The annotation workflow is described in Supplementary Figure 1.
Data analysis	BUSCO, Ortho-MCL, TransDecoder, CAFE, VESPA computational pipeline (as described, including ProtTest 3, MrBayes, and PAML), ClustalX, RPHAST, David Functional Annotation, Ingenuity Pathway Analysis.

For manuscripts utilizing custom algorithms or software that are central to the research but not yet described in published literature, software must be made available to editors and reviewers. We strongly encourage code deposition in a community repository (e.g. GitHub). See the Nature Portfolio guidelines for submitting code & software for further information.

Data

Policy information about availability of data

All manuscripts must include a data availability statement. This statement should provide the following information, where applicable:

- Accession codes, unique identifiers, or web links for publicly available datasets
- A description of any restrictions on data availability
- For clinical datasets or third party data, please ensure that the statement adheres to our policy

The sequence datasets generated and analysed in the current study are publicly available. The assembly is deposited into the National Center for Biotechnology Information Genbank Assembly database under accession # APMU00000000. Raw transcriptome sequences used for genome annotation are deposited into the National Center for Biotechnology Information Sequence Read Archive under accession # PRJNA474945. The genome annotation (doi:10.6084/m9.figshare.16654993) and source data for presented figures (doi:10.6084/m9.figshare.16655062.v1) are available on FigShare. There are no restrictions on data availability. All other data are available on request from the corresponding author.

Field-specific reporting

Please select the one below that is the best fit for your research. If you are not sure, read the appropriate sections before making your selection.

☒ Life sciences ☐ Behavioural & social sciences ☐ Ecological, evolutionary & environmental sciences

For a reference copy of the document with all sections, see nature.com/documents/nr-reporting-summary-flat.pdf

Life sciences study design

All studies must disclose on these points even when the disclosure is negative.

Sample size	Samples sizes were restricted by the number of seal samples available in tissue archives, and the number of fresh carcasses found in our study area during our deployment to Antarctica.
Data exclusions	No data were purposefully excluded. Some assays (e.g. qPCR have varied sample sizes due to limited amounts of cDNA available from a given tissue sample.
Replication	We relied on biological replicates for functional follow up experiments to compare differences between species. No replication was performed for genome construction or annotation.
Randomization	Samples were allocated into experimental groups by species.
Blinding	Blinding was not relevant to this study as we did not provide an experimental treatment to live subjects.

Reporting for specific materials, systems and methods

We require information from authors about some types of materials, experimental systems and methods used in many studies. Here, indicate whether each material, system or method listed is relevant to your study. If you are not sure if a list item applies to your research, read the appropriate section before selecting a response.

Materials & experimental systems

n/a	Involved in the study	
☒	☐	Antibodies
☒	☐	Eukaryotic cell lines
☒	☐	Palaeontology and archaeology
☐	☒	Animals and other organisms
☒	☐	Human research participants
☒	☐	Clinical data
☒	☐	Dual use research of concern

Methods

n/a	Involved in the study	
☒	☐	ChIP-seq
☒	☐	Flow cytometry
☒	☐	MRI-based neuroimaging

Animals and other organisms

Policy information about studies involving animals; ARRIVE guidelines recommended for reporting animal research

Laboratory animals	This study did not involve laboratory animals.
Wild animals	Tissue samples were collected at necropsy from Weddell seals (Leptonychotes weddellii) in Erebus Bay, Antarctica, then snap-frozen for transport. Genomic DNA was extracted from the liver of one female Weddell seal, and RNA samples for transcriptome sequencing were collected from the heart (left ventricle), muscle (longissimus dorsi), lung and placenta from other Weddell seals in the region (DNA extraction was made from a single tissue, n=4 adult seals). All Weddell seal handling and tissue collection were conducted under scientific authorizations from the National Marine Fisheries Service (NMFS 87-1851 & 19439) and the Antarctic Conservation Act. All tissue collection procedures with live animals were approved by the Massachusetts General Hospital IACUC. For gene expression analyses, we compared adult Weddell seal liver (n=3-4) and brain (cerebral cortex, n=3) to tissues collected from sheep (cerebral cortex n=4-6, liver n=4) (Ovis aries). Serum was analyzed from free-ranging Weddell seal pups (3 male, 2 female) juveniles (2 male, 3 female), and adults (2 male, 5 post-weaning females, 8 non-reproductive females), and wild harbor seals (5 adult males, obtained from a different study under authorization NMFS 18662). Seal samples were compared against a commercially purchased serum sample from monkey, rat, horse, sheep, and dog. Total triglycerides were also assayed in the livers and cerebral cortex of adult Weddell seals (n=5 liver, n=6 brain) and sheep (n=4 liver, n=4 brain).
Field-collected samples	Sample were collected in field, however were snap frozen for transport to the lab upon collection. Therefore, no housing information is relevant.
Ethics oversight	Massachusetts General Hospital IACUC, National Marine Fisheries Service

Note that full information on the approval of the study protocol must also be provided in the manuscript.
